# Diastolic dysfunction in patients with Fontan palliation is associated with death and heart transplantation

**DOI:** 10.1016/j.jocmr.2025.101961

**Published:** 2025-09-17

**Authors:** Edward H. Hardison, Christopher C. Henderson, Rachel K. Duncan, Kristen George-Durrett, James C. Slaughter, Ryan D. Byrne, Joshua D. Chew, Benjamin P. Frischhertz, David A. Parra, Angela J. Weingarten, Jonathan H. Soslow, Daniel E. Clark

**Affiliations:** a Division of Pediatric Cardiology, Primary Children’s Hospital at the University of Utah, Salt Lake City, Utah, USA; b Division of Pediatric Cardiology, Monroe Carrell Jr. Children’s Hospital at Vanderbilt, Nashville, Tennessee, USA; c Department of Biostatistics, Vanderbilt University Medical Center, Nashville, Tennessee, USA; d Division of Cardiovascular Medicine, Vanderbilt University Medical Center, Nashville, Tennessee, USA; e Department of Medicine, Stanford University School of Medicine, Palo Alto, California, USA

**Keywords:** Fontan, Diastolic dysfunction, Cardiac magnetic resonance imaging, Adult congenital heart disease, Single ventricle

## Abstract

**Background:**

Adults with Fontan failure (FF) have variable presentations and are often diagnosed late. Reliable predictors of FF are unknown. Diastolic dysfunction may be associated with adverse outcomes late after Fontan palliation.

**Methods:**

Fontan patients were compared to healthy controls. FF was defined as death, transplant, diagnosis of protein-losing enteropathy, peak VO_2_ <50% predicted, or new diuretic requirement. All phases in the short-axis plane were contoured to calculate filling and ejection curves. The following variables were measured by cardiovascular magnetic resonance (CMR): peak filling rate (PFR), peak ejection rate (PER), PFR and PER indexed to end diastolic volume (EDV), time to PFR (tPFR), and time to PER (tPER).

**Results:**

Compared to healthy controls (N = 96), the Fontan group (N = 98) had worse diastolic function as evidenced by decreased PFR and PFR/EDV and increased tPFR. Patients with FF (N = 39) had similar ventricular systolic function and volumetrics to the Fontan subjects without failure (NF; N = 59). PFR/EDV was significantly reduced, and indexed common ventricular mass was significantly higher among FF patients with the most severe adverse outcomes of death or heart transplantation. The prevalence of late gadolinium enhancement was higher in the FF cohort than the NF cohort.

**Conclusion:**

CMR can identify diastolic dysfunction in the Fontan population. Patients with Fontan circulation who died or had a combined outcome of death or transplant had worse diastolic function by CMR compared to Fontan patients without death or transplant.

## Graphical abstract

**Figure:**
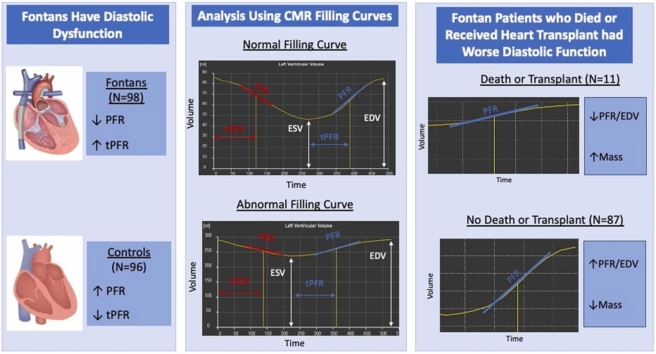

## 1 Background

Fontan palliation has steadily improved the survival of patients with single ventricle (SV) anatomy. The current estimated 30-year survival rate after the Fontan operation is 85% [1].

The hallmarks of Fontan physiology are systemic venous hypertension and low cardiac output [2]. These physiological consequences result in decreased exertional tolerance for patients with Fontan circulation, for which there are limited studied therapeutic interventions [3], [4]. Heart failure is the leading cause of death among adults with congenital heart disease and adults with Fontan circulation [5]. Morbidity is significant in early adulthood, with nearly half of patients reporting heart failure symptoms and only 41% remaining free from a serious adverse event by age 40 [6].

Due to the heterogeneity of Fontan failure (FF) phenotypes [7], early signs of FF can be a diagnostic challenge, and subtle symptoms of functional decline can go under-recognized by both providers and patients [8]. There is a growing need to find reliable indicators of FF to allow for early intervention. This is particularly important as increased time from the diagnosis of FF to heart transplant (HT) referral is associated with worse post-transplant outcomes.[5], [8], [9].

Diastolic dysfunction has been proposed as a subtype of FF and may also be a precursor to systolic heart failure [8]. Diastolic function is particularly important in SV physiology, as elevated filling pressures can directly affect cardiac output and impact long-term survival [1], [8], [10]. Diastolic dysfunction also causes increased Fontan pressures, leading to worse extra-cardiac disease in Fontan patients (such as Fontan-associated liver disease and renal disease), as well as the development of veno-venous collaterals and subsequent cyanosis. Some studies suggest that diastolic dysfunction may play a larger role than inadequate preload or elevated pulmonary vascular resistance in the long-term functional status of patients with Fontan palliation [11], [12]. Limited data currently exist on how to assess diastolic dysfunction in Fontan physiology noninvasively [8], [11]. Although some echocardiographic parameters—such as the atrioventricular systolic to diastolic ratio—have been shown to correlate with invasive pressure measurements in SV patients, conventional echocardiography remains an unreliable method for assessing diastolic function in SV patients [13], [14], [15]. Cardiovascular magnetic resonance imaging (CMR) is emerging as a reliable way to provide information on ventricular performance in patients with Fontan circulation, and recent studies have postulated that CMR could provide a better understanding of progressing diastolic dysfunction [1], [8], [16], [17], [18].

We hypothesized that patients with Fontan palliation would have diastolic dysfunction based on CMR filling curves compared to healthy controls and that patients with FF would have worse single ventricular filling and ejection than Fontan patients without FF (NF). To our knowledge, the present study is the first to investigate diastolic dysfunction using filling parameters in SV patients with CMR.

## 2 Methods

### 2.1 Study patient population

This study was approved by the Vanderbilt Institutional Review Board. We conducted a single-center, retrospective cohort study from April 2007 to June 2022 comparing patients with Fontan palliation to healthy controls to assess differences in CMR-derived indices of diastolic function. Clinical and demographic information was retrospectively collected at the most recent cardiology encounter prior to CMR and last follow-up and stored in an electronic, password-encrypted platform (research electronic data capture [REDCap]) [19].

Patients with Fontan palliation were categorized by the presence of FF or absence of Fontan failure (NF) at the last clinical follow-up. Fontan failure was defined a priori as the following: death, transplantation, durable mechanical circulatory support, lymphatic complications (protein-losing enteropathy and/or plastic bronchitis), peak VO_2_ <50% predicted on cardiopulmonary exercise stress testing, and/or a new standing diuretic requirement after formal evaluation by a cardiologist, according to previously published definitions [7], [20]. Major adverse cardiovascular events (MACEs) were defined as the presence of heart transplantation or death. A subgroup analysis was also performed on the patients with Fontan failure who experienced death or transplant. Atrioventricular valve regurgitation was defined subjectively based on the CMR report. Relevant cardiac catheterization data were included if the catheterization was performed within one year of CMR.

The cohort of healthy controls was derived from two sources as follows: 1) a group of patients 21 years old and younger with a normal CMR study as defined by normal biventricular size and systolic function without evidence of late gadolinium enhancement that were identified retrospectively, and 2) prospectively identified subjects, 21 years old and younger, who were enrolled as healthy volunteers to determine normal parametric mapping values for the magnets. For further details, please see the prior manuscript detailing this cohort [21].

### 2.2 CMR protocol and postprocessing

All subjects underwent CMR on a 1.5T system (Siemens Avanto or Avanto Fit magnet, Siemens Healthineers, Erlangen, Germany or a Philips Intera, Philips Healthcare, Best, The Netherlands). The CMR protocol consisted of cine balanced steady-state free precession (bSSFP) imaging to calculate systemic ventricular volumes and function and common ventricular volumes and function, with a standard temporal resolution of 25–30 phases per cardiac cycle. Intravenous gadolinium contrast (gadobutrol, Gadavist, Bayer Healthcare, Berlin, Germany, at a dose of 0.15 mmol/kg or Magnevist at a dose of 0.2 mmol/kg) was administered through a peripheral intravenous line according to clinical indications. Late gadolinium enhancement (LGE) was performed using segmented inversion recovery with the inversion time optimized for myocardial nulling and single-shot phase-sensitive inversion recovery (inversion time of 300 ms) in standard long- and short-axis planes.

**Fig. 1:**
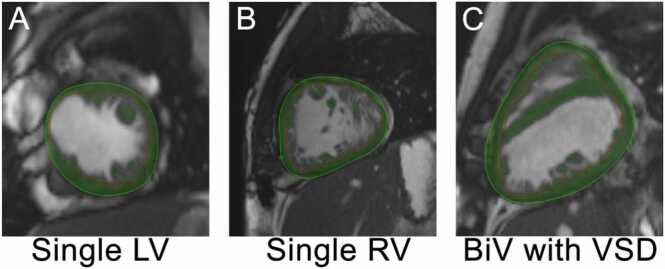
Example of ventricular contouring of a RV dominant, LV dominant, and 2-V images with thresholding. All short-axis phases were contoured using MASSk thresholding within QMass to generate ventricular filling and ejection curves. Contours were performed on the patient’s dominant, systemic ventricle. In the case of a large VSD, which resulted in both ventricles contributing to cardiac output, the entire ventricular mass was contoured. *LV* left ventricle, *RV* right ventricle, *BiV* biventricular, *VSD* ventricular septal defect

**Fig. 2:**
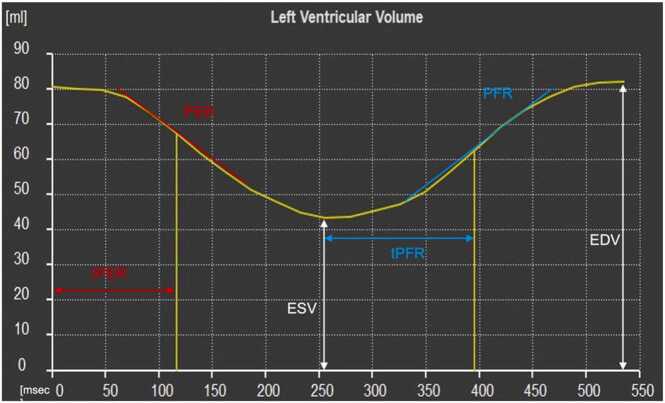
Left ventricular filling curve of a patient with Fontan physiology. *tPER* time to peak ejection rate, *PER* peak ejection rate, *ESV* end systolic volume, *tPFR* time to peak filling rate, *PFR* peak filling rate, *EDV* end diastolic volume

CMR postprocessing was performed blinded to all clinical data. Ventricular volumes and function were calculated using QMass (MedisSuite 2.1, Medis, Leiden, Netherlands). The presence or absence of LGE was annotated, and when present, LGE was quantified using the full-width half-maximum methodology. For volumetric and functional assessment, endocardial contours were performed on the patient’s dominant, systemic ventricle. In the case of a large ventricular septal defect, which resulted in both ventricles contributing to cardiac output, the entire ventricular mass was contoured (Fig. 1). All short-axis phases were contoured using MASSk thresholding within QMass to remove trabeculations and, in the cases of a large ventricular septal defect, the interventricular septum. Apical slices with partial volume averaging and any basal slices without ventricular myocardium throughout the cardiac cycle were removed from analysis as per our laboratory’s protocol [21]. Once all phases were contoured, QMass automatically generated ventricular filling and ejection curves and identified the peak ejection and filling rates. The following variables were included in the analysis: PFR, PER, PFR and PER indexed to the end diastolic volume of the single ventricle (PFR/EDV), time to peak filling rate (tPFR), time to peak ejection rate (tPER), and tPFR and tPER indexed to the RR interval (ms). Fig. 2 demonstrates an example filling curve adapted from Kikano and colleagues [22].

Figure adapted from Kikano and colleagues [22].

### 2.3 Statistical analysis

Categorical variables were compared using Pearson’s chi-squared test or Fisher’s exact test and reported as frequencies and percentages. Continuous variables were compared using the Mann–Whitney U test and reported as the mean and standard deviation. Effect sizes were calculated using rank-biserial correlation. Values of 0.1, 0.3, and 0.5 were considered to represent small, medium, and large effects, respectively. Statistical analysis was performed using STATA (version 15, Stata Corporation, College Station, Texas). To correct for differences in age between the control and Fontan group, a multivariable logistic regression analysis was performed with diagnosis as the outcome measure and age, body surface area, sex, and filling and ejection metrics as the predictors.

## 3 Results

### 3.1 Fontan patients versus controls

#### 3.1.1 Demographics

The demographic data of the Fontan and control groups are summarized in Table 1. At the time of data analysis, 116 patients with Fontan palliation had received a CMR at our institution from April 2007 to June 2022. Seventeen patients were excluded due to the inability to accurately assess volumetrics (9 due to excessive artifact, 6 due to the absence of short-axis cines, 1 terminated early due to patient agitation, and 1 due to suboptimal myocardial border detection). One additional patient was excluded because their CMR was after heart transplantation. Thus, CMRs for 98 patients with Fontan palliation were analyzed. There were 96 patients in the control group. The Fontan patients were older than the controls (19.0 vs. 14.5 years); there was no difference in sex. The median time from Fontan palliation to CMR in the Fontan group was 14 years (IQR, 11–21). Nineteen Fontan patients (19.4%) had a MACE. Thirty-nine Fontan patients (39.8%) had FF.

**Table 1:** Demographics of All Patients with Fontan Palliation and Controls^a^

	All Controls (N = 96)	All Fontan Subjects (N = 98)	Fontan Subjects Without Failure (N = 59)	Fontan Subjects With Failure (N = 39)
Age in years at time of CMR, median (IQR)	14.47 (11.8–18.3)	19.0 (14.0–26.0)	16.0 (13.0–23.0)	23.0 (19.0–30.5)
Time in years between Fontan and CMR, median (IQR)	-	14.4 (11.0–20.8)	12.4 (10.7–17.2)	17.8 (13.5–23.5)
Time in years between Fontan Failure and CMR, median (IQR)	-	-	-	2.8 (0.4–7.7) N = 38
Female, N (%)	44.8	41 (41.8)	22 (37.3)	19 (48.7)
Race, N (%)				
Asian	2 (2.1)	2 (2.0)	2 (3.4)	0 (0.0)
Black or African American	10 (10.4)	6 (6.1)	3 (5.1)	3 (7.7)
White	63 (65.6)	83 (84.7)	48 (81.4)	35 (89.7)
Not Reported	1 (1.0)	7 (7.1)	6 (10.2)	1 (2.6)
Ethnicity, N (%)				
Hispanic or Latino	-	9 (9.2)	6 (10.2)	3 (7.7)
Not Hispanic or Latino	-	86 (87.8)	51 (86.4)	35 (89.7)
Not Reported	96 (100)	3 (3.1)	2 (3.4)	1 (2.6)
BMI, median (IQR)	22.1 (18.4−26.1)	24.3 (20.8−28.3)	23.1 (18.7−27.4)	26.5 (22.2−30.5)
BSA, median (IQR)	1.7 (1.4−1.99)	1.8 (1.6−2.0)	1.8 (1.5−2.0)	1.87 (1.6−2.0)
Systemic Ventricle, N (%)				
Right	-	43 (43.9)	26 (44.1)	17 (44.6)
Left	-	54 (55.1)	32 (54.2)	22 (56.4)
Indeterminate	-	1 (1.0)	1 (1.7)	0(0)
Total MACE, N (%)	0.0	9 (9.2)	0	9 (23.1)
HT, N (%)	0.0	4 (4.1)	0	4 (10.3)
Death, N (%)	0.0	7 (7.1)	0	7 (17.9)

*CMR* cardiovascular magnetic resonance, *IQR* interquartile range, *BMI* body-mass index, *BSA* body surface area, *MACE* major adverse cardiovascular events, *HT* heart transplant.

a Data are numbers (%) of cases or medians (interquartile range).

#### 3.1.2 Volumetrics

The volumetric results of the Fontan palliation and control groups are listed in Table 2**.**

**Table 2:** CMR Volumetric Data of All Patients With Fontan Palliation and Controls^a^

	All Fontans (N = 98)	All Controls (N = 96)	*p*
EF, %	50.0 (43.0−53.7)	61.0 (58.0−65.0)	<0.001
EDV, mL	166.1 (133.0−228.0)	141.0 (112.5−170.0)	<0.001
EDVi, mL/m2	100.5 (80.3−124.0)	81.9 (75.0−90.5)	<0.001
ESV, mL	85.4 (59.3−116.0)	54.0 (40.0−65.0)	<0.001
ESVi, mL/m2	51.9 (40.5−65.8)	31.2 (27.0−35.1)	<0.001
PFR, mL/s	251.8 (206.6−297.2)	379.7 (292.2−457.8)	<0.001
tPFR, ms	149.3 (125.9−184.1)	126.2 (110.5−139.4)	<0.001
PFR/EDV, /s	2.0 (1.6−2.6)	3.3 (2.9−3.9)	<0.001

*EF* ejection fraction, *EDV* end-diastolic volume, *EDVi* indexed end-diastolic volune, *ESV* end systolic volume, *ESVi* indexed end systolic volume, *PFR* peak filling rate, *tPFR* time to peak filling rate.

a Data are presented as medians (interquartile range).

#### 3.1.3 Systolic function

Volumetric measures of systolic function included ejection fraction (EF), PER, and tPER. EF was significantly decreased in the Fontan group compared to the control group. PER trended towards being slower in the Fontan group than in the control group (median 299.1 mL/s, IQR 249.1–373.1 mL/s vs. 340.4, 251.1–424.3 mL/s, p = 0.203). The PER indexed to EDV (PER/EDV) was slower in the Fontan group than in the control group (2.5, 2.1–3.0 /s vs. 3.0, 2.7–3.2 /s, p<0.001). The tPER was longer in the Fontan group than in the control group (148.2, 129.1–179.1 ms vs. 124.5, 109.3–146.0 /s, p<0.001). Multivariable regression analysis demonstrated that PER (standardized effect size *t=*3.90, p<0.001), PER/EDV (*t=*3.96, p<0.001), and tPER (*t*=−4.63, p<0.001) were significantly different between Fontan and controls.

#### 3.1.4 Diastolic function

The PFR was significantly slower in the Fontan group than in the control group. Similarly, the PFR indexed to EDV (PFR/EDV) was lower in the Fontan group than in the control group. The tPFR was longer in the Fontan group than in the control group. We also investigated the correlation of volumetric parameters of diastolic dysfunction in the Fontan group (PFR, PFR/EDV, and tPFR) with other CMR parameters. There was a significant and moderate positive correlation between PFR and EDV (Spearman rho 0.364, p<0.001) and EDVi (Spearman rho 0.302, p = 0.003). This positive correlation remained when PFR was indexed to RR interval (tPFR/RR interval and EDV, Spearman rho 0.302, p = 0.003; tPFR/RR and EDVi, Spearman rho 0.206, p = 0.043). There was also a significant and moderate correlation between PFR/EDV and EF (Spearman rho 0.362, p<0.001). Multivariable regression analysis demonstrated that PFR *(t*=9.21*,* p<0.001)*,* PFR/EDV (*t*=9.27*,* p<0.001), and tPFR (*t=*−3.28, p = 0.001) were significantly worse in the Fontan group compared with the control group when corrected for age and BSA.

### 3.2 Right versus left systemic ventricle

A subgroup analysis was performed on volumetrics of the patients in the Fontan group with a clearly defined systemic ventricle, the results of which are summarized in Table 3. Patients with a systemic left ventricle (LV) had a higher EF, while those with a systemic right ventricle (RV) had a larger EDV and ESV. Patients with a systemic RV also had a significantly lower PFR/EDV than those with a systemic LV. There was no significant difference in PER, tPER, PFR, or tPFR between the two groups.

**Table 3:** Comparison of CMR Volumetrics in Patients in the Fontan Group With a Systemic Left or a Systemic Right Ventricle

	Systemic Right Ventricle (N=43)	Systemic Left Ventricle (N=54)	*p*
EF, %	45.5 (38.0–50.0)	52.3 (48.2–55.0)	**<0.001**
EDV, mL	186.4 (145.8–263.0)	141.3 (112.0–184.9)	**<0.001**
EDVi, mL/m2	112.0 (97.1–135.5)	91.7 (68.8–108.3)	**<0.001**
ESV, mL	106.0 (80.0–148.7)	71.0 (49.0–94.5)	**<0.001**
ESVi, mL/m2	61.4 (47.4–78.4)	44.7 (29.3–53.7)	**<0.001**
PFR, mL/s	257.7 (205.3–311.7)	242.5 (206.6–281.7)	0.276
tPFR, ms	154.8 (123.9–221.4)	144.2 (126.4–172.3)	0.189
PFR/EDV, /s	1.8 (1.4–2.5)^a^	2.3 (1.8–2.7)	**0.022**

Bold values indicate statistical significance.

*EF* ejection fraction, *EDV* end-diastolic volume, *EDVi* indexed end-diastolic volune, *ESV* end systolic volume, *ESVi* indexed end systolic volume, *PFR* peak filling rate, *tPFR* time to peak filling rate.

a Data are presented as medians (interquartile range).

### 3.3 Fontan failure versus no Fontan failure

#### 3.3.1 Demographics

Demographic data of the NF group (N = 59) compared to the FF group (N = 39) are also summarized in Table 1. The time of Fontan failure diagnosis was unavailable in one patient (diuretic initiation date was not listed). Dates of Fontan failure were extracted from chart review. The NF group was younger at the time of CMR (16.0 vs. 23.0 years) and had a shorter duration of time from Fontan palliation to CMR than the FF group (12.4 vs. 17.8 years). The NF group had a smaller proportion of female patients than the FF group (37.3% vs. 48.7%), and the systemic ventricle in both groups was predominantly the left ventricle. MACE occurred at a median of 20.7 years (IQR 13.7–27.0) after Fontan. The FF group had a higher incidence of MACE (23.1%) than the NF group (0%). Of the patients with the most severe outcomes of death or transplant, there was no significant difference in systemic ventricular morphology (63.6% RV vs. 36.4% LV, p = 0.171). Of the 7 patients who died, 3 had a systemic LV and 4 had a systemic RV. Of the 11 patients who had the outcome of death or transplant, 4 had a systemic LV and 7 had a systemic RV.

#### 3.3.2 Volumetrics

Subgroup analysis was stratified by FF, death, and the composite outcome of death or transplant (Table 4). There was no significant difference in parameters of either systolic or diastolic function between the NF and FF groups.

**Table 4:** Comparison of CMR Volumetrics in Patients With Fontan Palliation and Subgroups^a^

	All Patients with Fontan Physiology (N = 98)				FF Subgroup Analysis							
					*Death*				*Death or Transplant*			
	No Failure (N = 59)	Fontan Failure (N = 39)	*r*	*p*	Death (N = 6)	No Death (N = 92)	*r*	*p*	Death or Transplant (N = 11)	No Death or Transplant (N = 87)	*r*	*p*
Common ventricular EF (%)	50.3 (45.0–53.0)	47.0 (38.0–54.0)	0.168	0.138	40.0 (27.6–58.0)	50.0 (44.4–53.3)	0.073	0.138	38.0 (31.8–54.0)	50.0 (44.6–53.3)	0.17	0.088
Common ventricular EDV (mL)	163.0 (128.2–228.0)	169.0 (133.3–230.8)	−0.048	0.651	230.8 (144.3–247.0)	167.2 (133.0–225.1)	−0.104	0.203	204.4 (145.5–247.0)	161.0 (128.2–225.1)	−0.17	0.095
Common ventricular EDVi (mL/m^2^)	101.1 (87.4–124.3)	99.6 (76.5–126.5)	0.058	0.511	108.3 (70.1–127.5)	100.3 (81.5–124.3)	0.018	0.767	108.3 (72.9–158.5)	99.2 (81.0–120.9)	−0.14	0.179
Common ventricular EDV/BSA^1.3^ (mL/m^2^)	89.0 (75.5–104.8)	83.3 (59.4–105.6)	0.093	0.312	84.6 (56.3–105.6)	86.5 (74.3–105.3)	0.062	0.882	89.7 (59.4–144.9)	84.4 (73.7–104.0)	−0.1	0.308
Common ventricular ESV (mL)	81.9 (56.0–112.0)	91.6 (61.2–127.0)	−0.104	0.342	102.0 (48.7–167.1)	84.8 (59.3–114.0)	−0.091	0.11	117.8 (78.1–167.1)	83.3 (56.0–112.0)	−0.180	0.071
Common ventricular ESVi (mL/m2)	51.3 (41.7–62.0)	52.3 (36.5–73.6)	−0.019	0.948	44.7 (22.7–79.2)	52.0 (40.6–64.1)	0.006	0.534	78.4 (42.0–97.8)	51.5 (40.2–62.1)	−0.14	0.154
PFR (mL/s)	254.4 (213.8–297.2)	246.5 (197.1–298.9)	0.08	0.382	255.7 (224.1–314.2)	251.7 (205.3–297.2)	−0.021	0.635	255.7 (224.1–298.9)	246.5 (205.1–297.2)	−0.05	0.649
tPFR (ms)	148.5 (129.6–186.3)	150.2 (110.5–179.6)	0.091	0.37	154.8 (84.5–224.8)	148.6 (125.9–184.1)	0.003	0.563	154.8 (109.7–224.8)	148.6 (125.9–184.1)	−0.01	0.942
tPFR/RR interval	0.20 (0.15–0.24)	0.21 (0.15–0.25)	−0.002	0.986	0.21 (0.10–0.29)	0.20 (0.15–0.24)	0.008	0.94	0.21 (0.10–0.29)	0.20 (0.15–0.24)	0.201	0.951
PFR/EDV (/s)	2.1 (1.6–2.7)	1.8 (1.4–2.6)	0.132	0.204	1.4 (1.3–1.7)	2.1 (1.6–2.7)	0.231	**0.028**	1.4 (1.3–2.4)	2.1 (1.6–2.7)	−0.13	0.051
PER (mL/s)	287.7 (244.9–363.3)	308.1 (249.2–391.2)	−0.092	0.362	318.7 (308.1–391.2)	287.7 (243.8–363.3)	−0.164	0.105	318.7 (298.0–423.3)	287.7 (244.9–362.9)	−0.07	0.19
tPER (ms)	148.5 (134.2–180.1)	141.8 (114.6–177.7)	0.16	0.113	154.8 (126.7–179.6)	148.1 (129.1–179.1)	0.002	0.984	158.8 (126.7–187.3)	147.4 (129.1–177.7)	0.002	0.461
tPER/RR interval	0.20 (0.17–0.21)	0.20 (0.16–0.21)	0.051	0.614	0.21 (0.12–0.24)	0.20 (0.16–0.21)	−0.017	0.863	0.19 (0.13–0.24)	0.20 (0.16–0.21)	0.194	0.987
PER/EDV (/s)	2.5 (2.2–2.8)	2.5 (1.9–3.1)	0.019	0.85	2.2 (1.7–3.0)	2.5 (2.1–3.0)	0.135	0.181	1.8 (0.7–3.0)	2.6 (2.2–3.0)	−0.23	0.054
Common ventricular mass/BSA (g/m^2^)	44.5 (34.5–55.5)	52.0 (40.0–68.0)	−0.192	0.063	67.0 (53.0–90.0)	46.0 (38.0–61.0)	−0.173	0.248	66.5 (53.0–80.0)	46.0 (38.0–60.5)	−0.23	**0.028**

Bold values indicate statistical significance.

*FF* Fontan failure, *EF* ejection fraction, *EDV* end-diastolic volume, *EDVi* indexed end-diastolic volune, *ESV* end systolic volume, *ESVi* indexed end systolic volume, *PER* peak ejection rate, *PFR* peak filling rate, *r* rank-biserial correlation effect size, *tPER* time to peak ejection rate, *tPFR* time to peak filling rate, *BSA* body surface area.

a Data are presented as numbers (%) of cases or medians (interquartile range).

**Fig. 3:**
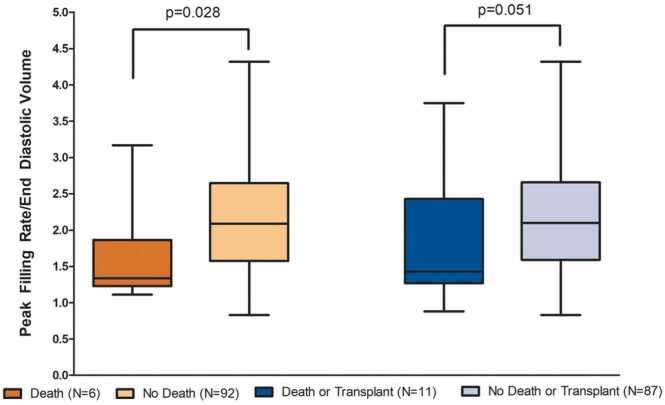
Comparison of peak filling rate/end diastolic volume (PFR/EDV) in patients with Fontan Palliation who experienced death or transplant. Whiskers represent minimum and maximum values; boxes represent Q1 and Q3; line represents median value. *PFR* peak filling rate, *EDV* end diastolic volume.

Patients who died (N = 6) had worse diastolic function than all other patients with Fontan palliation, with a smaller PFR/EDV. PFR, tPFR, and tPFR/RR were not significantly different between the two groups. Patients with a combined outcome of death or heart transplant (N = 11) also had worse diastolic function than all other patients with Fontan palliation (N = 87), indicated by a smaller PFR/EDV in the death or transplant group (Fig. 3).

AVVR was also investigated. There was no significant difference in measures of diastolic function between patients with less than moderate AVVR and those with moderate or more AVVR (median tPFR 148.6 ms vs. 150.2 ms, p = 0.480; PFR/EDV 2.1 /s vs. 1.8 /s, p = 0.354). Four patients had coarctation (3 in the FF group, 1 in the NF group). PFR/EDV trended higher in the coarctation group, but this was not significant (2.8 /s v. 2.1 /s, p = 0.07).

#### 3.3.3 Common ventricular mass

**Fig. 4:**
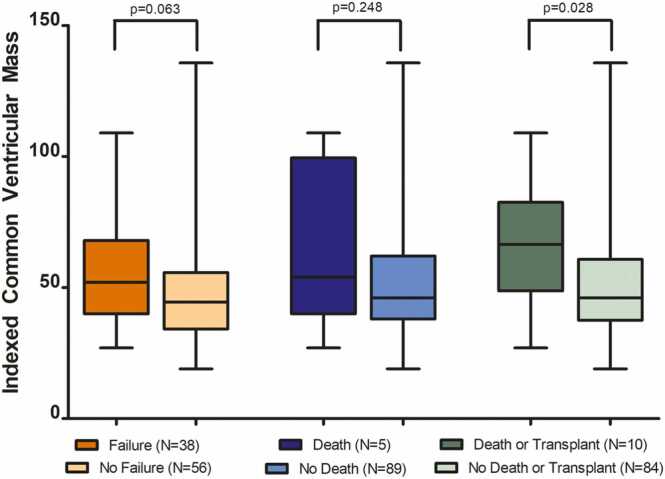
Comparison of indexed common ventricular mass in patients with Fontan Palliation with and without FF and those who experienced death or transplant. Whiskers represent minimum and maximum values; boxes represent Q1 and Q3; line represents median value. *FF* Fontan failure.

Common ventricular mass data were available for 94 patients (Fig. 4**)**. Common ventricular mass indexed to BSA trended toward higher values in the FF group compared to the NF group (52.0, 40.0–68.0 g/m^2^ vs. 44.5, 34.5–55.5 g/m^2^, p = 0.063). Patients who died trended toward increased indexed common ventricular mass compared to those who did not (67.0, 53.0–90.0 g/m^2^ vs. 46.0, 38.0–61.0 g/m^2^, p = 0.248), although this difference did not reach statistical significance. Patients with a combined outcome of death or transplant had increased indexed common ventricular mass compared with those without an outcome of death or transplant (66.5, 53.0–80.0 g/m^2^ vs. 46.0, 38.0–60.5 g/m^2^, p = 0.028).

#### 3.3.4 Late gadolinium enhancement

Of the 98 Fontan patients in our cohort, 66 patients had LGE analysis performed during their CMR. LGE was more prevalent among those with FF vs. NF (50% vs. 22.5%, p = 0.021). There was no significant difference in the amount of LGE, when present, between the two groups. Results are shown in Table 5**.**

**Table 5:** Comparison of Presence of Late Gadolinium Enhancement and Percentage of Late Gadolinium Enhancement in the CMRs of Patients With Fontan Palliation and Subgroup^a^

	No Failure (N = 40/59)	Failure (N = 26/39)	*p*	No Death (N = 63/92)	Death (N = 3/6)	*p*	No Death or Transplant (N = 60/87)	Death or Transplant (N = 6/11)	*p*
Patients with LGE	9 (22.5%)	13 (50.0%)	**0.021**	20 (21.7%)	2 (66.7%)	0.596	19 (31.7%)	3 (50.0%)	0.392
% LGE (median, IQR)	3.6 (2.4–6.3)	3.0 (2.1–9.7)	0.789	3.6 (2.3–6.3)	5.9 (2.0–9.7)	0.909	3.5 (2.2–6.3)	9.7 (2.0–11.2)	0.534

Bold values indicate statistical significance.

*LGE* late gadolinium enhancement, *IQR* interquartile range.

a Data are presented as number (%) of cases or medians (interquartile range).

#### 3.3.5 Cardiac catheterization

Twenty-three patients in our Fontan cohort underwent cardiac catheterization within 1 year of their CMR. Correlation coefficients for hemodynamics and CMR parameters are displayed in Table 6 while comparisons of hemodynamics between FF and outcome subgroups are demonstrated in Table 7**.** The pulmonary capillary wedge pressure (PCWP), end diastolic pressure (EDP), and Fontan pressure all correlated moderately with PER and PFR. Increased pulmonary vascular resistance (PVR) was associated with the outcome of death or transplant. All other invasive hemodynamic measurements were not statistically significant.

**Table 6:** Correlation of Cardiac Catheterization Hemodynamics With CMR Parameters^a^

	PCWP (mmHg)	EDP (mmHg)	Fontan Pressure (mmHg)	PVR (indexed Woods units)
PER (mL/s)	0.484 (**p=0.017**)	0.520 (**p=0.009**)	0.474 (**p=0.019**)	−0.040 (p=0.867)
tPER (ms)	−0.220 (p=0.302)	−0.256 (p=0.228)	−0.300 (p=0.154)	0.342 (p=0.141)
PER/EDV (/s)	0.034 (p=0.876)	0.238 (p=0.263)	0.079 (p=0.712)	−0.139 (p=0.560)
PFR (mL/s)	0.511 (**p=0.011**)	0.480 (**p=0.018**)	0.526 (**p=0.008**)	−0.018 (p=0.940)
tPFR (ms)	0.190 (p=0.373)	0.092 (p=0.669)	−0.060 (p=0.781)	0.030 (p=0.899)
PFR/EDV (/s)	0.048 (p=0.822)	0.248 (p=0.242)	0.090 (p=0.676)	−0.180 (p=0.448)
Common ventricular mass/BSA (g/m^2^)	0.201 (p=0.338)	0.167 (p=0.443)	0.062 (p=0.778)	0.026 (p=0.915)

Bold values indicate statistical significance.

*EDV* end-diastolic volume, *PER* peak ejection rate, *PFR* peak filling rate, *tPFR* time to peak filling rate, *BSA* body surface area.

a Data are presented as Spearman rho values.

**Table 7:** Comparison of Cardiac Catheterization Hemodynamics Between the Fontan Palliation Group and Subgroups^a^

	No Failure (N=10/59)	Fontan Failure (N=13/39)	*p*	Death (N=1/6)	No Death (N=22/92)	*p*	Death or Transplant (N=3/11)	No Death or Transplant (N=60/87)	*p*
EDP (mmHg)	9.0 (8.0–11.0)	8.0 (6.0–10.0)	0.778	12.0 (12.0–12.0)	8.0 (6.0–10.0)	-	8.0 (8.0–12.0)	9.0 (6.0–10.0)	0.782
RPA (mmHg)	12.5 (11.0–14.0)	11.0 (9.0–16.0)	0.390	Not obtained	12.0 (11.0–16.0)	-	10.5 (10.0–11.0)	12.5 (11.0–16.0)	0.251
LPA (mmHg)	12.0 (12.0–14.0)	12.0 (9.5–16.0)	0.616	Not obtained	12.0 (10.0–16.0)	-	12.0 (11.0–13.0)	12.0 (10.0–16.0)	0.809
PCWP (mmHg)	8.0 (7.0–10.0)	8.0 (6.0–10.0)	0.999	13.0 (13.0–13.0)	8.0 (6.0–10.0)	-	9.0 (7.0–13.0)	8.0 (6.0–10.0)	0.540
PVR (iWU)	1.5 (1.5–1.8)	2.0 (1.4–2.7)	0.533	9.2 (9.2–9.2)	1.7 (1.5–2.3)	-	2.7 (2.5–9.2)	1.6 (1.5–2.0)	**0.021**
Glenn Pressure (mmHg)	13.0 (12.0–15.0)	12.0 (10.0–18.0)	0.641	22.0 (22.0–22.0)	13.0 (11.0–16.0)	-	12.0 (11.0–22.0)	13.0 (12.0–16.0)	0.964
Fontan Pressure (mmHg)	13.0 (12.0–16.0)	13.0 (11.0–16.0)	0.492	21.0 (21.0–21.0)	13.0 (11.0–16.0)	-	12.0 (11.0–21.0)	13.0 (11.0–16.0)	0.927

Bold values indicate statistical significance.

*EDP* end-diastolic pressure, *RPA* right pulmonary artery, *LPA* left pulmonary artery, *PCWP* pulmonary capillary wedge pressure, *PVR* pulmonary vascular resistance, *iWU* indexed Wood units.

a Data are presented as medians (interquartile range).

## 4 Discussion

To our knowledge, our study is the first to utilize CMR parameters to demonstrate the extent of diastolic dysfunction in adults with Fontan circulation as well as their implication in Fontan outcomes. Fontan patients have a higher incidence of diastolic dysfunction than healthy controls. CMR indices of diastolic dysfunction, specifically PFR/EDV and indexed common ventricular mass, have the strongest correlation with death or transplant among patients with Fontan palliation, independent of systemic ventricular morphology. Although CMR metrics of systolic and diastolic function were not significantly different between Fontan patients with and without FF in this cohort of predominantly young adults, the diastolic parameters of PFR/EDV and indexed common ventricular mass were associated with increased incidence of MACE. The findings support our hypothesis that patients with Fontan palliation have ubiquitous diastolic dysfunction based on CMR filling curves compared to healthy controls of a similar age with biventricular circulation. In our cohort, patients with Fontan palliation had a significantly longer tPFR, reduced PFR, and lower PFR/EDV.

Diastolic dysfunction in patients with Fontan physiology is a topic of emerging relevance in the literature [1], [8], [10], [11], [12]. Chowdhury and colleagues recently used echocardiography to demonstrate that diastolic dysfunction was associated with the clinical phenotype of heart failure with preserved EF in the SV population [23]. However, historically, noninvasive measures of diastolic dysfunction have proven challenging and unreliable. Typical echocardiographic parameters used to assess congestion (left atrial enlargement, estimated right ventricular systolic pressure, and subpulmonary chamber enlargement) are not findings that can be assessed in Fontan patients, emphasizing the need for alternative imaging modalities to fully adjudicate diastolic ventricular performance in the Fontan circulation [24].

Ventricular volumetric data have previously been shown to be useful measurements of diastolic dysfunction in the biventricular population [15], [20]. When comparing all Fontan patients with and without FF, there was no significant difference in volumetric data or diastolic function. However, patients with the most significant outcomes—death, or the combined outcome of death or transplant—did have significantly worse SV filling. These findings are likely reflective of the heterogeneity of Fontan failure and may suggest that differences in diastolic function by CMR emerge as patients are nearing the end stages of their Fontan palliation.

Previous studies have found ventricular dilation to be predictive of time to death or listing for transplant in the Fontan population [16], [25]. In our cohort, there was no significant difference in ventricular dilation as a continuous variable (calculated both as EDV/BSA^1.3^ and EDV/BSA) between the NF and FF groups or in subgroup analysis with outcomes of death or transplant. This may be related to differences in the cohorts, with our cohort having higher BSAs, a higher predominance of systemic LV morphology, and a higher percentage of female patients. Decreased ventricular volume may play a role in the assessment of diastolic function, and it is unlikely that any measure is truly preload- or afterload-independent; thus, the PFR/EDV may be the best marker and somewhat independent from preload.

Given the different properties of a systemic ventricle in the Fontan patient, we analyzed volumetrics between patients with a systemic LV and those with a systemic RV. Our results are consistent with prior studies demonstrating that systemic RV volumes are larger than LV volumes [26]. Although there was no difference in filling and ejection measurements in these two groups, there was a significant difference between PFR/EDV and PER/EDV between RV and LV. However, this was likely driven by the significant difference in EDV and ESV between RV and LV.

LGE has also been shown to be a useful metric in the evaluation of diastolic dysfunction in the biventricular population [17]. In our cohort, there was a significant difference in the presence of LGE in the FF group compared to the NF group. However, there were no statistically significant differences in LGE between those with death and/or transplant compared to those without MACE.

Hemodynamic indices among Fontan patients have repeatedly been shown to have poor association with adverse outcomes in this cohort [27], [28], [29]. A few significant correlations were found between available hemodynamic data from cardiac catheterization and CMR volumetrics. PCWP, EDP, and Fontan pressure all correlated moderately and positively with PER and PFR. Interestingly, these seem to have the opposite correlation from that expected. This could be due to the small sample size and a more significant effect of volume status on PCWP and EDP. It is also likely that ventricular compliance in patients with Fontan circulation is dynamic, and that the rate of filling may be a more accurate measure of diastolic function. If so, then resting filling pressure may not fully assess diastolic function, which is supported by recent studies on exercise catheterization [30]. Catheterization data (obtained only at rest per institutional practice) did not correlate with PFR/EDV. Given the small number of patients who underwent cardiac catheterization within a year of CMR, these data should be examined further in a larger cohort.

Ventricular mass evaluated by CMR can also provide useful diastolic assessment [17]. In our cohort, indexed common ventricular mass was consistently higher in patients who died or underwent transplantation. These findings are similar to those previously demonstrated in the literature and suggest that diastolic dysfunction is a feature in Fontan patients with the worst clinical outcomes [25]. Ventricular mass did not have any significant correlation with catheterization data.

Systolic function was also abnormal in the Fontan group compared to controls. Fontan patients had significantly decreased EF, tPER, and PER/EDV compared to controls. However, no systolic metrics were significant between the FF and NF groups or associated with the more severe outcomes of death or transplant.

## 5 Limitations

As a single-center, retrospective cohort study, our results cannot be generalized to be predictive of FF; rather, they are associations. Additionally, given our small cohort size, we are unable to determine a useful cutoff value of PFR/EDV associated with FF. In addition, this study was underpowered to perform multivariable regression within the Fontan group. A larger cohort would be helpful to validate the results of the current investigation, to determine clinically relevant cutoffs, and to perform multivariable regression.

CMR as a modality also introduces bias, as some Fontan patients were unable to undergo CMR either due to devices (e.g., epicardial pacing systems) or intolerance (e.g., patient habitus, anxiety in scanner, or disability).

We also used an established definition of FF that is broad and includes patients with a diuretic requirement as prescribed by a cardiologist. One could argue that the addition of a maintenance diuretic does not constitute a heart failure phenotype; however, this is both a previously accepted definition of FF in the literature and a more sensitive definition of FF.

## 6 Conclusions

Patients with Fontan palliation had significant systolic and diastolic dysfunction compared to healthy controls. Patients with clinically defined FF did not have a significant difference in systolic or diastolic metrics from those without FF. However, patients with Fontan palliation who died or had a combined outcome of death or transplant had evidence of worse diastolic function compared to those with Fontan palliation without failure. Future prospective studies with a larger cohort are necessary to understand the prognostic implications of abnormal diastolic CMR parameters such as PFR/EDV and indexed common ventricular mass in adult Fontan outcomes.

## Funding

Not applicable.

## Author contributions

Edward H. Hardison Writing – review & editing, Writing – original draft, Visualization, Validation, Supervision, Resources, Project administration, Methodology, Investigation, Formal analysis, Data curation, Conceptualization^a,^*^,1,2^ (nedhardison@gmail.com), Christopher C. Henderson Writing – review & editing, Data curation^b^, Rachel K. Duncan Writing – review & editing, Data curation^b^, Kristen George-Durrett Resources, Data curation^b^, James C. Slaughter Writing – review & editing, Formal analysis^c^, Ryan D. Byrne Writing – review & editing^b,d^, Joshua D. Chew Writing – review & editing^b^, Benjamin P. Frischhertz Writing – review & editing^b,d^, David A. Parra Writing – review & editing^b^, Angela J. Weingarten Writing – review & editing^b,d^, Jonathan H. Soslow Writing – review & editing, Writing – original draft, Methodology, Investigation, Formal analysis, Data curation, Conceptualization^b^, Daniel E. Clark Writing – review & editing, Writing – original draft, Supervision, Methodology, Investigation, Formal analysis, Data curation, Conceptualization.

## Ethics approval and consent

This study was approved by the Vanderbilt Institutional Review Board. Informed consent was waived since this study was retrospective.

## Consent for publication

Not applicable.

## Declaration of competing interests

The authors declare that they have no known competing financial interests or personal relationships that could have appeared to influence the work reported in this paper.

## Data Availability

The datasets used and/or analyzed during the current study are available from the corresponding author on reasonable request.

## References

[1] Rychik J., Atz A.M., Celermajer D.S., Deal B.J., Gatzoulis M.A., Gewillig M.H. (2019). Evaluation and management of the child and adult with Fontan circulation: a scientific statement from the American Heart Association. Circulation.

[2] Rychik J. (2016). The relentless effects of the Fontan paradox. Semin Thorac Cardiovasc Surg Pediatr Card Surg Annu.

[3] Khiabani R.H., Whitehead K.W., Han D., Restrepo M., Tang E., Bethel J. (2015). Exercise capacity in single-ventricle patients after Fontan correlates with haemodynamic energy loss in TCPC. Heart.

[4] Goldberg D.J., Zak V., Goldstein B.H., Schumacher K.R., Rhodes J., Penny D.J. (2020). Results of the FUEL trial. Circulation.

[5] Menachem J.N., Schlendorf K.H., Mazurek J.A., Bichell D.P., Brinkley D.M., Frischhertz B.P. (2020). Advanced heart failure in adults with congenital heart disease. JACC Heart Fail.

[6] Dennis M., Zannino D., du Plessis K., Bullock A., Disney P.J.S., Radford D.J. (2018). Clinical outcomes in adolescents and adults after the Fontan procedure. J Am Coll Cardiol.

[7] Book W.M., Gerardin J., Saraf A., Marie Valente A., Rodriguez F. (2016). Clinical phenotypes of Fontan failure: implications for management. Congenit Heart Dis.

[8] Budts W., Ravekes W.J., Danford D.A., Kutty S. (2020). Diastolic heart failure in patients with the Fontan circulation: a review. JAMA Cardiol.

[9] Lewis M.J., Reardon L.C., Aboulhosn J., Haeffele C., Chen S., Kim Y. (2023). Morbidity and mortality in adult Fontan patients after heart or combined heart-liver transplantation. J Am Coll Cardiol.

[10] Kutty S., Jacobs M.L., Thompson W.R., Danford D.A. (2020). Fontan circulation of the next generation: why it’s necessary, what it might look like. JAHA.

[11] Cheung Y.F. (2000). Serial assessment of left ventricular diastolic function after Fontan procedure. Heart.

[12] Goldstein B.H., Connor C.E., Gooding L., Rocchini A.P. (2010). Relation of systemic venous return, pulmonary vascular resistance, and diastolic dysfunction to exercise capacity in patients with single ventricle receiving Fontan palliation. Am J Cardiol.

[13] Margossian R., Schwartz M.L., Prakash A., Wruck L., Colan S.D., Atz A.M. (2009). Comparison of echocardiographic and cardiac magnetic resonance imaging measurements of functional single ventricular volumes, mass, and ejection fraction (from the Pediatric Heart Network Fontan Cross-Sectional Study)††A list of participating institutions and investigators appears in the Appendix. Am J Cardiol.

[14] Cordina R., Ministeri M., Baby-Narayan S.V., Ladouceur M., Celermajer D.S., Gatzoulis M.A. (2018). Evaluation of the relationship between ventricular end-diastolic pressure and echocardiographic measures of diastolic function in adults with a Fontan circulation. Int J Cardiol.

[15] Miranda W.R., Warnes C.A., Connolly H.M., Taggart N.W., O’Leary P.W., Oh J.K. (2019). Echo-Doppler assessment of ventricular filling pressures in adult Fontan patients. Int J Cardiol.

[16] Rathod R.H., Prakash A., Kim Y.Y., Germanakis I.E., Powell A.J., Gauvreau K. (2014). Cardiac magnetic resonance parameters predict transplantation-free survival in patients with Fontan circulation. Circ Cardiovasc Imaging.

[17] Chamsi-Pasha M.A., Zhan Y., Debs D., Shah D.J. (2020). CMR in the evaluation of diastolic dysfunction and phenotyping of HFpEF. JACC Cardiovasc Imaging.

[18] Kawaji K., Codella N.C.F., Prince M.R., Chu C.W., Shakoor A., LaBounty T.M. (2009). Automated segmentation of routine clinical cardiac magnetic resonance imaging for assessment of left ventricular diastolic dysfunction. Circ Cardiovasc Imaging.

[19] Harris P.A., Taylor R., Thielke R., Payne J., Gonzalez N., Conde J.G. (2009). Research electronic data capture (REDCap)—a metadata-driven methodology and workflow process for providing translational research informatics support. J Biomed Inform.

[20] Byrne R.D., Weingarten A.J., Clark D.E., Huang S., Perri R.E., Scanga A.E. (2019). More than the heart: hepatic, renal, and cardiac dysfunction in adult Fontan patients. Congenit Heart Dis.

[21] Henderson C.C., George-Durrett K., Kikano S., Slaughter J.C., Chew J.D., Parra D. (2023). Reference data for left ventricular filling and atrial function in children using cardiovascular magnetic resonance. J Cardiovasc Magn Reson.

[22] Kikano S.D., Weingarten A., Sunthankar S.D., McEachern W., George-Durrett K., Parra D.A. (2023). Association of cardiovascular magnetic resonance diastolic indices with arrhythmia in repaired Tetralogy of Fallot. J Cardiovasc Magn Reson.

[23] Chowdhury S.M., Graham E.M., Taylor C.L., Savage A., McHugh K.E., Gaydos S. (2022). Diastolic dysfunction with preserved ejection fraction after the Fontan procedure. JAHA.

[24] Redfield M.M., Borlaug B.A. (2023). Heart failure with preserved ejection fraction: a review. JAMA.

[25] Meyer S.L., St. Clair N., Powell A.J., Geva T., Rathod R.H. (2021). Integrated clinical and magnetic resonance imaging assessments late after Fontan operation. J Am Coll Cardiol.

[26] Alsaied T., Li R., Christopher A., Fogel M., Slesnick T.C., Krishnamurthy R. (2024). Characterization and Z-score calculation of cardiac MRI parameters in patients after the Fontan operation. A Fontan outcome registry using CMR examinations (FORCE) study. J Cardiovasc Magn Reson.

[27] Mori M., Hebson C., Shioda K., Elder R.W., Kogon B.E., Rodriguez F.H. (2016). Catheter-measured hemodynamics of adult Fontan circulation: associations with adverse event and end-organ dysfunctions. Congenit Heart Dis.

[28] Kiesewetter C.H., Sheron V., Vettukattill J.J., Hacking N., Stedman B., Millward-Sadler H. (2007). Hepatic changes in the failing Fontan circulation. Heart.

[29] Kaulitz R., Luhmer I., Bergmann F., Rodeck B., Hausdorf G. (1997). Sequelae after modified Fontan operation: postoperative haemodynamic data and organ function. Heart.

[30] Miranda W.R., Borlaug B.A., Jain C.C., Anderson J.H., Hagler D.J., Connolly H.M. (2023). Exercise-induced changes in pulmonary artery wedge pressure in adults post-Fontan versus heart failure with preserved ejection fraction and non-cardiac dyspnoea. Eur J Heart Fail.

